# Serum Lipoprotein(a) and High-Sensitivity C-reactive Protein Correlate With Somatic Parameters Including MLPA Subgroups in Children With Prader-Willi Syndrome

**DOI:** 10.1210/jendso/bvaf082

**Published:** 2025-05-08

**Authors:** Pritam Biswas, Sudipta Banerjee, Paromita Bhattacharya, Krishanu Mukhoti, Moulibrata Sarkar, Subhankar Chowdhury, Pranab Kumar Sahana

**Affiliations:** Department of Endocrinology, IPGME&R, Kolkata 700020, India; Department of Endocrinology, IPGME&R, Kolkata 700020, India; Genetic Service Unit, BRIC-NIBMG, PG-Polyclinic, Kolkata 700020, India; Department of Respiratory Medicine, IPGME&R, Kolkata 700020, India; Department of Radiodiagnosis, IPGME&R, Kolkata 700020, India; Department of Endocrinology, IPGME&R, Kolkata 700020, India; Department of Endocrinology, IPGME&R, Kolkata 700020, India

**Keywords:** Prader-Willi-Syndrome, lipoprotein-(a), hs-CRP, fat-free mass index, deletion pathology

## Abstract

**Context:**

Prader-Willi syndrome (PWS) is 1 of the most common monochromosomal (15q11-13) causes of polygenic syndromic childhood obesity.

**Objectives:**

We primarily compare and correlate serum lipoprotein(a) [Lp(a)], high-sensitivity C-reactive protein (hs-CRP), and baseline clinical characteristics of genetically confirmed children with PWS at their GH treatment-naïve stage to their control groups. Secondary objectives were to correlate serum Lp(a) and hs-CRP concentration to multiplex ligation-dependent probe amplification subgroups, body composition indices, sleep apnea parameters, and hepatic shear-stress by 2-dimensional shear wave elastography in children with PWS.

**Methods:**

A total of 32 genetically confirmed PWS children (age 5 to 18 years), 20 simple obesity children, and 20 healthy children as age-matched control groups were studied for the primary and secondary study objectives.

**Results:**

Lp(a) was higher in the study group as compared to the control group (*P*  *<* .0001), but we found no difference between the control groups (*P* = .9680).In addition, no correlation was detected in Lp(a) levels in the study population with respect to their body weight, body mass index, and waist circumference. hs-CRP levels were also higher in the study population compared to both control groups (*P* = .0962; *P* < .0001); in contrast, Lp(a) differed significantly between the control groups (*P* = .002). Lower fat-free mass index (FFMI) correlated with higher levels of serum Lp(a) (*r* = −0.5525; *P* = .001), whereas FFMI was not correlated with hs-CRP levels in PWS children (*P* = .657). Based on genomic subtypes, patients with PWS were divided into deletion and nondeletion genetic subgroups. We found significantly altered levels of Lp(a), hs-CRP, fat-free mass, and sleep apnea parameters, particularly in the deletion subgroup.

**Conclusion:**

Serum Lp(a) as well as hs-CRP stand out to be the core independent risk factors along with their strong correlation with the other study parameters, which necessitates the role of future targeted therapeutics in PWS, especially in deletion pathology. Thus, genetic subtyping during diagnostic confirmation endorses further prognostic elaboration.

Since its initial identification in 1956, Prader-Willi syndrome (PWS) has been considered a disorder primarily affecting children, characterized by obesity, short stature, cryptorchidism, and intellectual disability [1]. Symptoms typically start to manifest during infancy [2]. Now it is recognized as a severe neurodevelopmental and complex endocrine disorder that includes cognitive disabilities and behavioral problems with multiphase clinical presentations [3, 4]. This disorder was the first to be identified as an imprinting genetic disorder [5]. Currently, there are 4 distinct genomic subtypes observed [6]: paternal deletion, maternal uniparental disomy, imprinting defects, and translocation. These subtypes could be clubbed as nondeletion genetic subgroups [7]. In our country, this disease is classified as rare, due to its estimated global prevalence of 1 in 20 000 to 30 000 newborns [8]. Despite significant advancements in multidisciplinary care, the reported morbidity and mortality rates range from 1.25% to 3.00% per year, which is 3 times higher than the rates observed in the general population [9, 10]. The majority of cases were associated with sudden cardiac death and respiratory failure. Serum lipoprotein(a) [Lp(a)] is recognized as an independent risk factor for premature atherosclerotic cardiovascular disease and aortic valvular disease [11, 12]. The serum high-sensitivity C-reactive protein (hs-CRP) level can serve as a reliable predictor of early coronary heart disease (CHD), even in individuals who are considered healthy. It has a greater potential for accurately assessing the risk of CHD and sudden cardiac death compared to serum cholesterol levels [13, 14]. Additionally, in children with high-risk characteristics, it can be used along with Lp(a) to screen for the risk of CHD and sudden cardiac death [14].

In our study, we primarily compare and correlate serum Lp(a) levels, hs-CRP levels, and baseline clinical characteristics of genetically confirmed children with PWS at their GH treatment-naïve stage to the age-matched control population of children with simple obesity and healthy children. Our secondary objectives were to correlate serum Lp(a) and hs-CRP concentration to genetic subgroups (deletion vs nondeletion), body composition indices, sleep apnea parameters, and hepatic shear stress by 2-dimensional shear wave elastography in children with PWS.

## Materials and Methods

This was an observational and descriptive hospital-based epidemiological study.

### Study Participants

Thirty-two children between 5 and 18 years of age with methylation-specific multiplex ligation-dependent probe amplification (MS-MLPA) genetic test-confirmed PWS were recruited by a nonprobability purposive sampling method. Twenty children with simple childhood obesity and another 20 healthy children were taken as 2 age-matched control population groups in the estimation of serum Lp(a) and hs-CRP, in addition to the clinical study variables, to test our primary objective. Children with simple obesity were defined by childhood obesity in the age range 5 to 18 years (according to the Indian Academy of Pediatrics growth charts) who had no monogenic or syndromic clinical associations and were genetically negative for PWS. Healthy children were defined as age-matched normal children with a body mass index (BMI) below the 85th percentile for age (Supplementary Fig. S1) [15].

The power of the study was calculated by G*Power 3.1.9.7 software, and the study was approved by the institutional ethics committee and research oversight committee of the Institute of Post Graduate Medical Education and Research, Kolkata, India (Letter No. IPGME&R/IEC/2023/403) in accordance with the code of ethics of the World Medical Association (Declaration of Helsinki) for experiments involving humans.

### Blood Collection and Sample Processing

A fast of 8 to 10 hours was recommended to the study participants before the collection of blood samples. Venous blood of 4 to 6 mL was collected in clot vials from each of the participants. Serum was separated by a centrifugation method at 825 g for 10 minutes at 4 °C; serum samples were then preserved in cryovials at – 80 °C for further biochemical assays.

Serum Lp(a) and hs-CRP were measured as per the manufacturer's protocol by the ELISA method with the sandwich-ELISA principle [Lp(a) kit RRID: AB_3678691, catalog no. E-EL-H0160, Elabscience Biotechnology, Inc., USA; sensitivity 0.14 ng/mL, detection range 0.23-15 ng/mL; hs-CRP kit RRID: AB_3678692, catalog no. E-EL-H5134, Elabscience Biotechnology Inc., USA; sensitivity 9.38 pg/mL, detection range 15.63-1000 pg/mL]. Assay repeatability of both Lp(a) and hs-CRP had a coefficient of variation of less than 10.

Clinically suspected study participants were genetically confirmed and subgrouped into deletion (copy number = 1; ratio = 0.5) and nondeletion (copy number = 2; ratio = 1.0) genetic categories following the MS-MLPA method (SALSA MLPA Probemix ME028 kit; SALSA HhaI enzyme and *coffalyser.Net* data analysis software, MRC Holland). Here, copy number variations and methylation status were measured in the 15q11-13 region of genomic DNA isolated from peripheral whole blood specimens.

Total cholesterol, triglycerides, high-density lipoprotein cholesterol (HDL-C), and low-density lipoprotein cholesterol were measured by an enzymatic method.

Among the secondary study variables, fat-free mass index (FFMI) and fat mass index (FMI) were estimated by an enCORE-based x-ray bone densitometer (GE Healthcare Lunar, USA); apnea hypopnea index (AHI) and oxygen desaturation percentage were measured by a Resmed ApneaLink device (ResScan v.6.0.1); and 2-dimensional shear wave elastography of the liver was estimated by a LOGIQ P9 ultrasound device (GE Healthcare Ultrasound Systems, USA).

Anthropometric measurements, including height, weight, BMI, and waist circumference, were recorded for all participants.

### Statistical Analysis

The normality of the continuous variables was tested by the D’Agostino and Pearson test. Data were expressed using median and interquartile range (IQR). An unpaired *t*-test was conducted for comparison of the 2 normally distributed groups and a Mann–Whitney *U* test for nonnormally distributed groups. Depending on the distribution, the correlation between 2 variables was represented using the Pearson or Spearman correlation coefficient. A *P-*value of less than .05 was considered statistically significant. GraphPad Prism (version 9) and SPSS 26 (trial version) were used to perform statistical analyses.

## Results

In the study cohort of 32 PWS children, 65.71% were males; 34.28% belonged to the lower middle class and 8.57% to the upper socioeconomic class based on the modified Kuppuswamy scale [16] (Supplementary Fig. S2) [15]. Twenty-five percent of children were from a rural background, and 74.28% came from urban or semiurban cities.

Common initial clinical presentations were history of neonatal-infantile hypotonia (100%), gross motor developmental delay (100%), eating disorders (93.1%), and speech and language delay (96.5%). Childhood-onset obesity, mostly a severe variant, was also present in 93.1%, and overweight was found in 6.89%. Other clinical manifestations at initial presentation are described in Supplementary Table S1 [15].

We summarize the relevant clinical and biochemical variables of the study participants along with the control populations in Table 1. Serum triglycerides, HDL-C, low-density lipoprotein cholesterol, and triglyceride-to-HDL-C ratio did not significantly differ among the study groups except serum non-HDL-C, which was significantly higher in children with PWS compared to children with simple obesity (*P* = .0457).

**Table 1.** Baseline clinical and biochemical study variables of study and control populations

	Study variables	PWS cases (A), M (IQR)	*P*-values (compare A and B)	Simple obesity controls (B), M (IQR)	*P-*values (compare A and C)	Healthy controls (C), M (IQR)	*P*-values (compare B and C)
1.	Age, years	10 (6.25-12.75)	.443	11 (7.25-16.25)	.8921	10 (7.25-11.75)	.2823
2.	Body weight, kg	46 (35.2-70.08)	.5166	43.95 (34.45-55.53)	**.0007**	26.80 (22.83-37.85)	**.0072**
3.	BMI, kg/m^2^	29.07 (27.45-32.53)	.2596	28.10 (24.84-32.27)	**<.0001**	17.16 (15.73-18.19)	**<.0001**
4.	Waist circumference, cm	70.60 (65.9-90.2)	.2101	67.3 (59.43-94.33)	**.0002**	63.10 (52.33-70.10)	**.0356**
5.	TG, mg/dL	130.50 (114.50-164.80)	.6916	125.50 (104-179.50)	.3519	128.0 (88.25-150)	.6538
6.	HDL, mg/dL	40.00 (35.00-42.00)	.9589	38.50 (34.25-44.25)	.4549	40.0 (36.50-42.0)	.4965
7.	TG/HDL	3.589 (2.893-4.601)	.6782	3.766 (2.513-4.485)	.1272	3.246 (2.813-3.938)	.2797
8.	LDL, mg/dL	74.00 (52.75-84.00)	.5980	69.00 (53.25-75.75)	.8850	74.0 (55.50-82.75)	.5687
9.	Non-HDL, mg/dL	120.00 (100-130.30)	**.0457**	100.0 (93-117)	.1026	100.0 (80-127.50)	.5565
10.	Lp(a), ng/mL	92 506 (IQR:11 724–114 904)	**<.0001**	2342 (1917–12 779)	**<.0001**	2699 (2047-8009)	.9680
11.	hs-CRP, ng/mL	287.35 (103.48-1353.30)	.0962	216.28 (35.78-341.71)	**<.0001**	24.85 (15.93-63.49)	**.002**

Statistically significant values were made bold font.

Abbreviations: BMI, body mass index; HDL, high-density lipoprotein; hs-CRP, high-sensitivity C-reactive protein; IQR, interquartile range; LDL, low-density lipoprotein; Lp(a), lipoprotein(a); PWS, Prader-Willi syndrome; TG, triglyceride.

Serum Lp(a) was significantly higher in the study group as compared to the simple obesity (*P* < .0001) and healthy control groups (*P* < .0001) (Table 1), but there was no statistically significant differences in serum Lp(a) levels between the control groups (*P* = .9680). In addition to the previous results, there was no significant correlation (Table 2) detected in serum Lp(a) levels in the study population with respect to their body weight, BMI, and waist circumference. Serum hs-CRP levels were also significantly higher in the study population compared to both control groups (*P* = .0962; *P* < .0001) (Table 1). In contrast to serum Lp(a), serum hs-CRP was higher in the simple obesity control group in comparison to the healthy control group (*P* = .002). Their body weight and BMI were not statistically correlated to the serum Lp(a) compared to the serum hs-CRP level (*P* = .002; P = .001), respectively. The body composition indices [FFMI (kg/m^2^) and FMI (kg/m^2^)] in our study cohort were altered from the age-specific cut-off references from non-Asian ethnicity [17]. Lower FFMI was correlated significantly with higher levels of serum Lp(a) (*r* = *−*0.5525; *P* = .001) (Supplementary Fig. S4) [15], whereas FFMI was not significantly associated with serum hs-CRP levels in PWS children (*P* = .657) (Table 2). In contrast to FFMI, FMI was found to have a positive correlation with serum hs-CRP levels (*r* = 0.419; *P* = .017) (Supplementary Fig. S5, Table 2) [15]. According to the age-dependent cut-off for sleep apnea [18-20] and after adjusting for polysomnography confounders, there was a 100% and 75% incidence of sleep apnea for children up to 12 years and older than 12 years, respectively. Median values of AHI and maximum oxygen desaturation were 11.95 events/hour (IQR: 7.275-18.380) and 86.00% (IQR: 74.25-89.75), respectively. AHI was found to have neutral effects on serum Lp(a) level alteration, but it had a statistically significant positive correlation with serum hs-CRP levels (*r* = 0.7964*; P* < .0001) (Table 2). In view of liver shear stress severity cut-offs for fibrosis stages (F0: 4.4 ± 0.6; F1: 5.6 ± 0.6; F2-3: 7.1 ± 0.7), the incidence rates of hepatic stiffness were 18.75% for F0, 12.5% for F1, and 68.75% for F2-F3 [21]. Alanine aminotransferase levels were strongly correlated with hepatic shear stress and shear wave velocity (*P* < .0001; Spearman correlation).

**Table 2.** Correlation of serum Lp(a) and hs-CRP levels with different somatic study parameters

	Serum Lp(a) level		Serum hs-CRP level	
	Correlation coefficient (r)	*P*-value	Correlation coefficient (r)	*P*-value
Body weight	0.168	.784	0.894	**.002**
BMI	0.245	.217	0.749	**.001**
Waist circumference	0.206	.266	0.358	**.047**
FMI	0.294	.101	0.419	**.017**
FFMI	−0.552	**.001**	0.081	.657
AHI	−0.241	.182	0.796	**<.0001**
2D-SWE	0.482	**.005**	0.416	**.017**

Statistically significant values were made bold font.

Abbreviations: 2D-SWE, 2-dimensional shear wave elastography; AHI, apnea hypopnea index; BMI, body mass index; FFMI, fat-free mass index; FMI, fat mass index; hs-CRP, high-sensitivity C-reactive protein; Lp(a), lipoprotein(a).

There were 62.5% deletion and 37.5% nondeletion MS-MLPA genetic subgroups among the PWS study cohort. Study participants with deletion pathology revealed significantly higher serum concentrations of Lp(a) as well as hs-CRP (Table 3). In the body composition analysis, FFMI was found to be higher in the deletion subgroup (*P* = .039), but the FMI was comparable between the genetic subgroups. The degree of sleep apnea was less affected in nondeletion PWS children (*P* = .026). Hepatic stiffness was comparable in both genetic subgroups (*P* = .781).

**Table 3.** Comparison of the study parameters between the 2 genetic subgroups (deletion vs nondeletion)

	Deletion subgroup, M (IQR)	Nondeletion subgroup, M (IQR)	*P-*value
Lp(a), ng/mL	101 540 (88 642–114 474)	12 197 (10 854–88 995)	**.020**
hs-CRP, ng/mL	614.72 (128.52-2677.91)	150.11 (27.14-596.82)	**.039**
FFMI, kg/,m^2^	8.47 (7.23-9.00)	9.79 (7.87-12.06)	**.039**
FMI, kg/m^2^	8.99 (7.02-10.79)	8.60 (6.17-10.78)	.863
AHI, events/hour	13.45 (9.20-23.35)	7.95 (3.45-13.53)	**.026**
2D-SWE, kPa	7.85 (6.27-9.27)	7.65 (4.57-10.55)	.781

Statistically significant values were made bold font.

Abbreviations: 2D-SWE, 2-dimensional shear wave elastography; AHI, apnea hypopnea index; FFMI, fat-free mass index; FMI, fat mass index; hs-CRP, high-sensitivity C-reactive protein; IQR, interquartile range; Lp(a), lipoprotein(a).

## Discussion

Age at diagnosis has a bimodal distribution, with the first peak in early childhood (younger than 4 years) and the second in the peripubertal period (10-13 years) [22]. In our study cohort, the average age of presentation was 10 years. Approximately 42% and 37% of children were 5 to 7 years and 12 to 18 years, respectively (Supplementary Fig. S3) [15]. Instead of an equal gender distribution [23], we found approximately a 1.9:1 male-to-female child ratio among the study participants. There is no national or global data regarding socioeconomic class with respect to the family living with the PWS child. We collected data regarding family income, education, and occupation per Indian evidence [16], and the most common socioeconomic class was found to be lower-middle (as per the modified Kuppuswamy classification). We also subcategorized our population according to their residential area. We found that the majority of the study participants lived in either urban or semiurban areas, which indicates the existence of social underawareness about childhood syndromic obesity as well as poor access to specialized healthcare systems for the rural population. Common initial clinical presentations were corroborative with the existing literature [24].

Serum non-HDL-C levels are higher in children with steatohepatitis than steatosis, suggesting increased cardiovascular disease risk. This may be a reflection of the higher prevalence of metabolic syndrome. Non-HDL-C had a positive association with histologic features of non-alcoholic steatohepatitis [25]. In our study, we found raised serum non-HDL-C in the PWS cohort compared to children with simple obesity (*P* = .0457), along with a considerable proportion of higher-grade hepatic fibrosis in the PWS children cohort, measured by 2D shear wave elastography. Butler et al revealed in their study that fasting plasma lipids did not significantly differ in PWS patients compared to the controls [26]. In another study, serum lipidomics analyses found that phosphatidylcholine and lysophosphatidylcholine were significantly low in PWS, whereas triacylglycerol was higher compared to the control population [27].

Serum Lp(a) levels in the study participants were significantly higher compared to both the simple obesity and healthy control groups (Table 1), and levels were not influenced by body mass (Table 2). Elevated serum levels of Lp(a) serve as an independent risk indicator for early-onset atherosclerotic cardiovascular disease and aortic valve stenosis in adults [28]. Lp(a) induces proinflammatory and proatherogenic effects. As much as 90% of the variability in Lp(a) plasma concentrations is genetically influenced. Lp(a) has become a valuable metric for risk assessment and familial screening in adults. The existing recommendations from the European Society of Cardiology and the European Atherosclerosis Society advocate for the measurement of Lp(a) in every individual at least once during their lifetime. The American Heart Association also endorsed the role of Lp(a) measurement in the pediatric population. Evidence suggests that Lp(a) may serve as a potential biomarker in clinical practice for assessing cardiovascular health and risk in children over 2 years of age and adolescents. Unlike adults, data regarding Lp(a) serum levels in children are scarce, and it remains uncertain whether Lp(a) screening in this population would similarly detect individuals at heightened risk of atherosclerotic cardiovascular disease. Timely identification and intervention at the proatherogenic state is essential for the primary prevention of chronic exposure of vascular walls to harmful lipoproteins, thereby averting the onset of atherosclerotic cardiovascular disease, which likely begins in childhood in PWD. There is increasing evidence that elevated Lp(a) levels are detectable from a young age [12]. In addition to the study variables discussed in this research, we found 77.14% with GH deficiency, 14.28% with secondary hypothyroidism, and 80.00% with hypogonadism during the process of routine evaluation of the PWS children cohort. Serum Lp(a) levels in the PWS children cohort were elevated compared to the controls; 81.48% had GH deficiency, 20.00% had secondary hypothyroidism, and 78.57% had hypogonadism. As GH treatment increases serum Lp(a) levels [29], we should be vigilant during the period of GH therapy in PWS children. In adults, there is evidence of a positive correlation between serum Lp(a) and hypogonadism [30], but there are no such studies available in children. Hypothyroidism may correlate with serum Lp(a) levels in the adult population [31]. Therefore, looking for changes in serum Lp(a) levels upon treatment with levothyroxine in hypothyroid PWS children could be a future avenue.

Children with cardiovascular risk factors had significantly higher serum levels of hs-CRP compared to the healthy population, and these levels positively correlated with BMI [14]. In our study cohort, serum hs-CRP was also significantly higher compared to the age-matched healthy control group but was comparable to the simple obesity controls (Table 1). Serum hs-CRP also had a strong correlation with body mass indices (Table 2). Butler et al stated a similar finding to our study result but with a C-reactive protein estimation instead of hs-CRP level [32]. Another study revealed that hs-CRP is lower in PWS compared to obese controls [33]. Thus, its level could be used in PWS children as a predictive marker for early cardiovascular disease in adulthood.

A 3-compartment body composition model divides body mass into fat mass, nonosseous lean body mass, and bone mass. Dual energy x-ray absorptiometry scan offers a near-accurate, quick, and precise means of this 3-compartment analysis. Compartment-specific body mass indices such as the FMI and FFMI have been proposed as more accurate indicators of adiposity compared to BMI [34]. We found altered body composition indices (FFMI, FMI) in our study participants compared to the references of age-specific values in non-Asian cohorts (White, Black, Hispanic), as currently there is no reference available for the Asian population [17]. FFMI was negatively correlated with serum Lp(a) (*r* = −0.5525; *P* = .001) compared to hs-CRP levels (*r*  *=* 0.419; *P* = .017), whereas FMI was positively correlated with serum hs-CRP only (*r* = 0.419; *P* = .017) (Table 2). Thus, low FFMI could be taken as an independent and early cardiovascular risk factor, similar to serum Lp(a), compared to FMI, in children diagnosed with PWS. We found a considerable number of PWS children suffering from obstructive sleep apnea based on age-specific diagnostic cut-offs [18] for AHI and oxygen desaturation level. Miller et al showed a similar incidence of sleep apnea in PWS children at baseline [20]. AHI had a statistically significant positive correlation with serum hs-CRP levels (*r* = 0.7964; *P* < .0001) compared to Lp(a). Therefore, body mass might have a role in the pathogenesis of obstructive sleep apnea in PWS children.

Our MS-MLPA genetic subgroup analysis between deletion and nondeletion pathology revealed a deletion predominance at around 62%, whereas in the existing literature, the deletion to nondeletion ratio is about 50% in neonates and 65% in the overall PWS population [24, 35]. We found significantly altered levels of serum biomarkers, fat-free mass, and sleep apnea, particularly in the deletion genetic subgroup. Thus, we could utilize genetic subgrouping as an independent prognostic predictor in the GH treatment-naïve PWS children along with its role in genetic counseling to the parents [36-39]. Coupaye et al described in their cohorts the effects of genetic subtypes on body anthropometries [39, 40].

One of the strengths of the study is all participants were genetically confirmed cases who were also GH-treatment naïve cases, which avoided confounding effects on the study variables. In addition, we recorded socioeconomic aspects of healthcare. Despite the low sample size in the study participants and control groups, the study has adequate statistical power [(1-β) = 0.9350; α = .05] in post hoc power analysis.

We also have certain study limitations; biomarkers were measured by the ELISA method not by liquid chromatography–mass spectrometry. We did not have regional normative ranges for some key study parameters; all study participants had minimal geographical variations and thus effects of ethnic variations studied.

Regarding future directions, in our institute, there is another ongoing longitudinal study in which the effects of standard-dose recombinant human GH therapy on these study parameters in PWS children will be tested and followed at 6- and 12-month intervals.

## Conclusion

Serum Lp(a) as well as hs-CRP stand out to be the core independent risk factors along with their strong correlation with other study parameters, which necessitates the role of future targeted therapeutics in PWS, especially in deletion genetic pathology. Thus, genetic subtyping during diagnostic confirmation endorses further prognostic elaboration. Due to higher awareness of the disease and access to tertiary facilities, in our study, an urban population supersedes the rural community in terms of childhood syndromic obesity healthcare, which underscores the need for improvement in early detection and risk stratification of childhood syndromic obesity through the clinical endpoints at a grassroots level.

## Data Availability

Data are available on request from the corresponding author. Supplementary data are available in the data repositories mentioned in the References.
